# Two-dimensional transition metal carbides and nitrides (MXenes) based biosensing and molecular imaging

**DOI:** 10.1515/nanoph-2022-0550

**Published:** 2022-11-14

**Authors:** Huiyu Liu, Xiaotong Xing, Yan Tan, Haifeng Dong

**Affiliations:** Marshall Laboratory of Biomedical Engineering, Research Center for Biosensor and Nanotheranostic, School of Biomedical Engineering, Health Science Center, Shenzhen University, Shenzhen 518060, China

**Keywords:** biosensors, microRNA detection, molecular imaging, MXenes, two-dimensional nanomaterials

## Abstract

As a “star material”, 2D transition metal carbides and/or nitrides (MXenes) have tremendous potential applications in biosensor development and molecular imaging. MXenes have a lot of advantages due to their large specific surface, excellent electrical conductivity, adjustable band gap, and easy modification. MXenes that immobilized with DNA strands, proteins, enzymes, or other bioluminescent materials on the surface, have been used to measure small molecules with extraordinary sensitivity and remarkable limit of detection. This review provides an overview of most recent development in the synthesis, fundamental properties, biosensing, and molecular imaging applications of MXenes. We focused on molecular detection through MXene-based electrochemical properties their challenges and novel opportunities of MXenes in biological applications. This article will provide a guide for researchers who are interested in the application of MXenes biosensors.

## 1 Introduction

**Figure 1:**
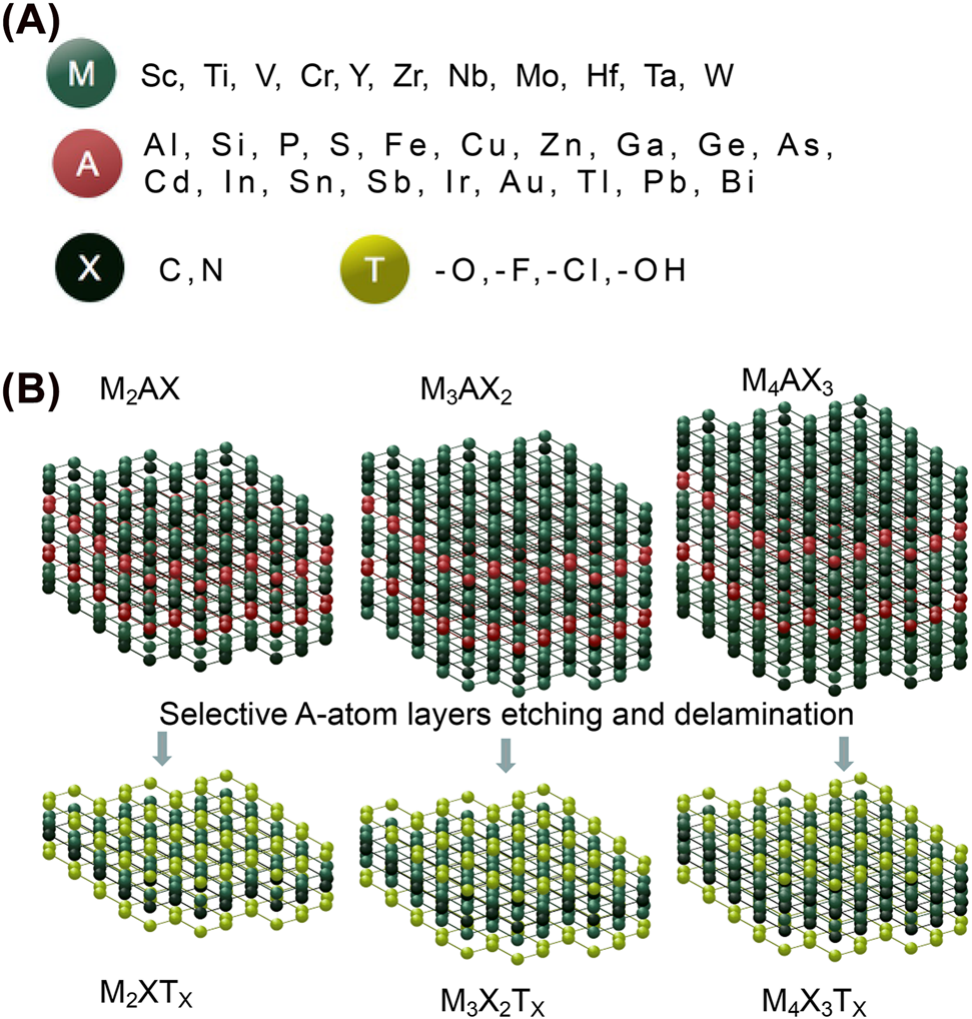
The elements and structures of MXenes. (A) Chemical formula of MXenes. Wherein M represents an early transition metal element (Sc, Ti, V, Cr, Y, Zr, Nb, Mo, Hf, Ta, W, etc.), A means a group element including Al, Si, P, S, Fe, Cu, Zn, Ga, Ge, As, Cd, In, Sn, Sb, Ir, Au, Tl, Pb, Bi, etc. X represents C or N. T represents a surface end group (–OH, –O, –F, etc.). (B) Etching processes from MAX to MXenes phases.

Since the two-dimensional (2D) graphene nanosheets were magnificently exfoliated from bulk graphite in 2004 [1, 2], 2D nanomaterials have attracted extensive interest and become one of the most dynamic research areas in nanotechnology. Etching the A element from the MAX phase of layered ceramic yielded 2D transition metal carbonitrides, generating a new class of nanomaterials termed MXene materials. The chemical formula of MXene is M_*n*+1_X_*n*_T_*x*_ [3, 4], wherein M represents an early transition metal element (Sc, Ti, V, Cr, Y, Zr, Nb, Mo, Hf, Ta, W, etc.), X represents C or N, and *n* = 1 to 4. T represents a surface end group (–OH, –O, –F, etc.) (Figure 1A).

MXene materials derived from MAX have become the most prominent family of 2D materials due to the diversity of MAX phase compositions and structures [5]. Gogotsi group successfully prepared Ti_3_C_2_ nanosheets by etching Ti_3_AlC_2_ with hydrofluoric acid (HF) at room temperature in 2011, which promoted progress in the synthesis and characterization of MXenes [6]. The structure and preparation of M_2_X, M_3_X_2_, and M_4_X_3_ are shown in Figure 1B [7].

**Scheme 1:**
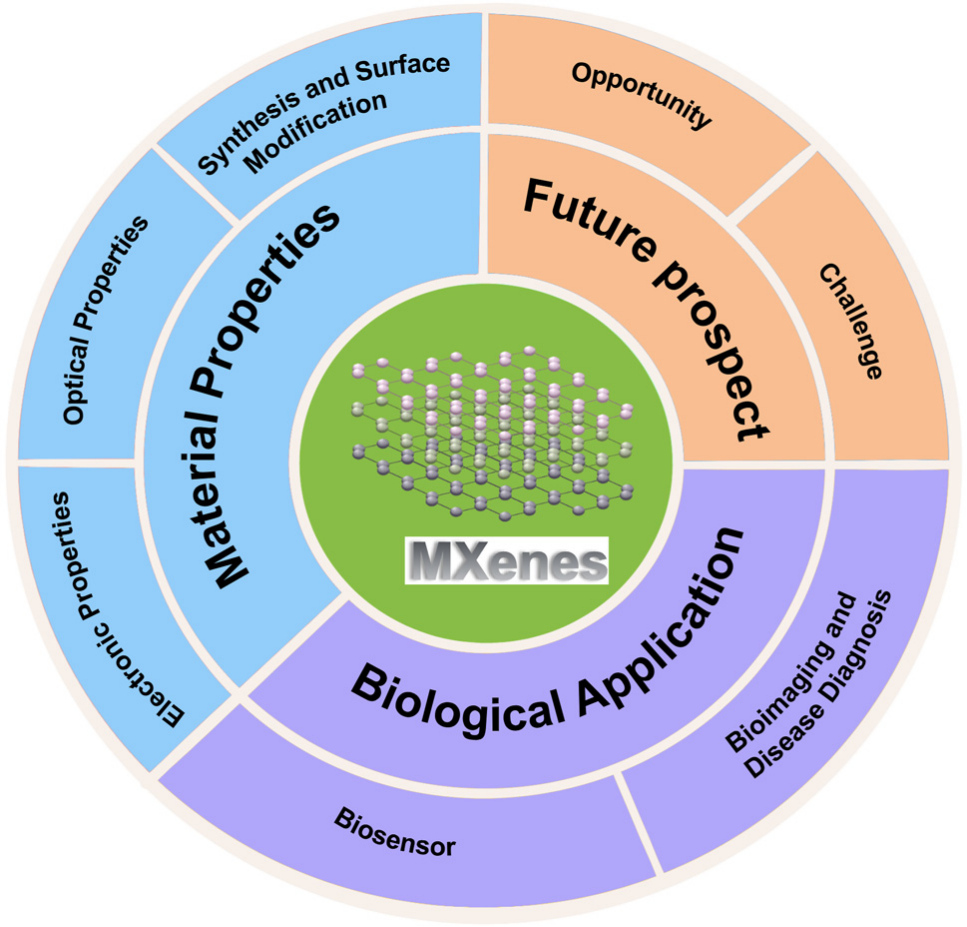
Overview of MXenes in this review.

Considering the research on MXene materials in our group, the goal of this review mainly focused on the summary of the latest progress in the field of biosensing using MXenes. We provide an overview of MXenes and describe the synthesis, properties, and surface modifications. Furthermore, we elucidate the MXenes-based detection of microRNA and other small molecules and biomedical imaging applications. Finally, we illustrate the challenges for MXenes-based analysis. This review will outline the significant developments of MXenes in molecular detection and bioimaging applications. It will also serve as a reference for researchers by highlighting notable instances of their application in microRNA detection (Scheme 1).

## 2 Properties of MXenes

MXenes have a lamellar structure with weak interlayer bonds and strong in-plane covalent bonds. Almost all of the atoms of MXenes are exposed after exfoliation (ultrathin form). The increased surface area of these MXenes materials considerably improves their chemical and physical reactivity, enabling drug loading and functional modification, and generating unique photonic, catalytic, magnetic, and electronic characteristics not found in bulk materials. MXenes are employed to construct innovative nanoplatforms with intriguing functions for optical imaging, photothermal imaging, biosensing, and targeted drug delivery. Thus, MXenes have great potential in biomedical applications [8, 9].

### 2.1 Electronic property

MXenes have either conductor or semiconductor properties due to their ordered arrangement of metal atoms. The various coating groups of MXenes produce distinct electron attraction capabilities, which profoundly influence their electrical properties. A significant influence on the metal layer’s electrical properties would occur from the imbalanced distribution of electron density caused by the increase in surface defects created during the synthesis of MXenes, which hinders the free flow of electrons [10]. MXenes have the capacity to produce active electrons and vacancies in response to specific stimuli (such as light excitation), and the interaction between MXene and surrounding environment is able to cause oxidative stress. The electrical conductivity can be accurately determined by measuring the current changing between the electrodes after binding to the testing species. A change in the local charge field initiates the signal accumulation which is reflected by material’s conductivity. The main advantage of MXenes as electronic biosensors is that the cross-sectional area is on the same spatial scale as the charge field of the nearby biomolecules, which serve as the smallest spatially limited target to sense electrical changes as a function of the measured conductivity [11]. Moreover, MXenes can produce active electrons under external stimuli such as heat or light.

### 2.2 Optical property

According to “first-principles density functional theory,” the calculations show that the optical band gap of MXenes can be changed by modifying the surface group compositions and contents. The linear optical properties (e.g., absorption, photoluminescence) and nonlinear optical properties (e.g., saturable absorption, nonlinear refractive index) highly depended on the structure [12]. Compared to the MXenes with the same thickness, the MAX phase absorbs more light and is transparent under visible light irradiation. Halim group established that MXenes films with larger intercalants were more transparent and less conductive [13]. The optoelectronic properties of the films may be modified by the electrochemical intercalation of the cations, exhibiting reversible transmission in the UV–Vis range, indicating the potential of MXenes to act as transparent conductors [14]. The UV–Vis spectrum shows a clear difference between MAX and MXene that MXene has significant absorption near 300 nm and a broad absorption near 800 nm, while the MAX showed weak absorption of at the range of 200–1000 nm [15, 16].

Nonlinear optics relate to the interplay between light and matter interactions in the nonlinear response of materials to electromagnetic fields. This phenomenon is crucial for laser optics, photonic devices, and optical communications applications. The unique photoluminescence lifetime of MXene nanomaterials can be directly used as a valuable probe for sensing and enabling live cell imaging [17]. Additionally, MXenes have potential to generate reactive oxygen species (ROS) under light irradiation, which can be designed as a platform for simultaneous photoacoustic imaging (PAI) and photodynamic therapy (PDT) [18]. However, the photothermal conversion mechanism of MXenes has not yet fully developed. Dong et al. speculated that MXenes like Ti_3_C_2_ have a localized surface plasmon resonance (LSPR) effect that similar to the gold nanoparticles [19]. It has the ability of photothermal conversion in the near-infrared (NIR) light region. Similarly, Wang’s group demonstrated that MXenes have photothermal conversion ability in NIR because of their excellent electromagnetic interference shielding and LSPR effect [20]. The Mo_2_Ti_2_C_3_T_*x*_ MXene material reported by Guo et al. exhibited excellent saturable absorption properties, with an enhanced 40% modulation depth observed within the double transition carbide compared to previously reported MXenes, and can be used as a passively Q-switched mid-infrared fiber laser for SAM [21].

## 3 Synthesis and surface modification of MXenes

### 3.1 Synthesis methods

The efficient synthesis of MXene is the basis for expanding the materials’ range of applications. MAX has a crystal structure, and “M” atoms form an octahedral-like structure. “X” atoms are filled in the gaps of the octahedron. “A” intercalated into the lamellae formed with M and X and finally obtained an M_*n*+1_AX_*n*_ structure. Primarily, the M–X bond belongs to the covalent bond and ionic bond. M–A and A–A belong to metallic bonds, and their bond energy is lower than that of the M–X bond, so the “A” atom is more active and easily stripped. The method for synthesizing MXenes is mainly a chemical etching method. Generally, MXenes materials are fabricated using two different methods: a top-down approach based on multilayer bulk flake exfoliation and a bottom-up approach to growing 2D flakes from their precursor salts. These synthesis methods endow various physical, surface, chemical, and electronic properties that can be used to perceive the connection between structural and functional properties [22]. In a top-down approach, thin layers of MXene films are exfoliated from their MAX phase precursors, primarily by mechanical exfoliation [23, 24]. Moreover, this is a liquid strip method with lower production costs and extended production capacity. The bottom-up approach depends on combining appropriate “metal-organic molecules” to make MXene films, mostly by metal-organic decomposition, chemical vapor deposition (CVD), wet chemistry, and other methods. Large areas of two-dimensional defect-free monolayer crystals can also be synthesized [25].

#### 3.1.1 Top-down method

The top-down method for MXenes preparation is selectively etching the A layer in MAX material. It is mainly divided into HF etching, fluoride etching, molten salt, alkali-assisted hydrothermal, and other methods. Because the bond between the M layer and the A layer of the MAX phase is a solid covalent or metallic bond, MXenes was synthesized from the MAX phase by selective etching of the A layer by temperature processing [26]. The smaller the force constant contributed by adjacent atoms to the A atom, the smaller the exfoliation energy and the more manageable the exfoliation. Electrons injected into the MAX phase lead to the elongation of the M–A bond, which further induces the MAX phase’s swelling and the layer’s exfoliation [27].

#### 3.1.2 HF etching

**Figure 2:**
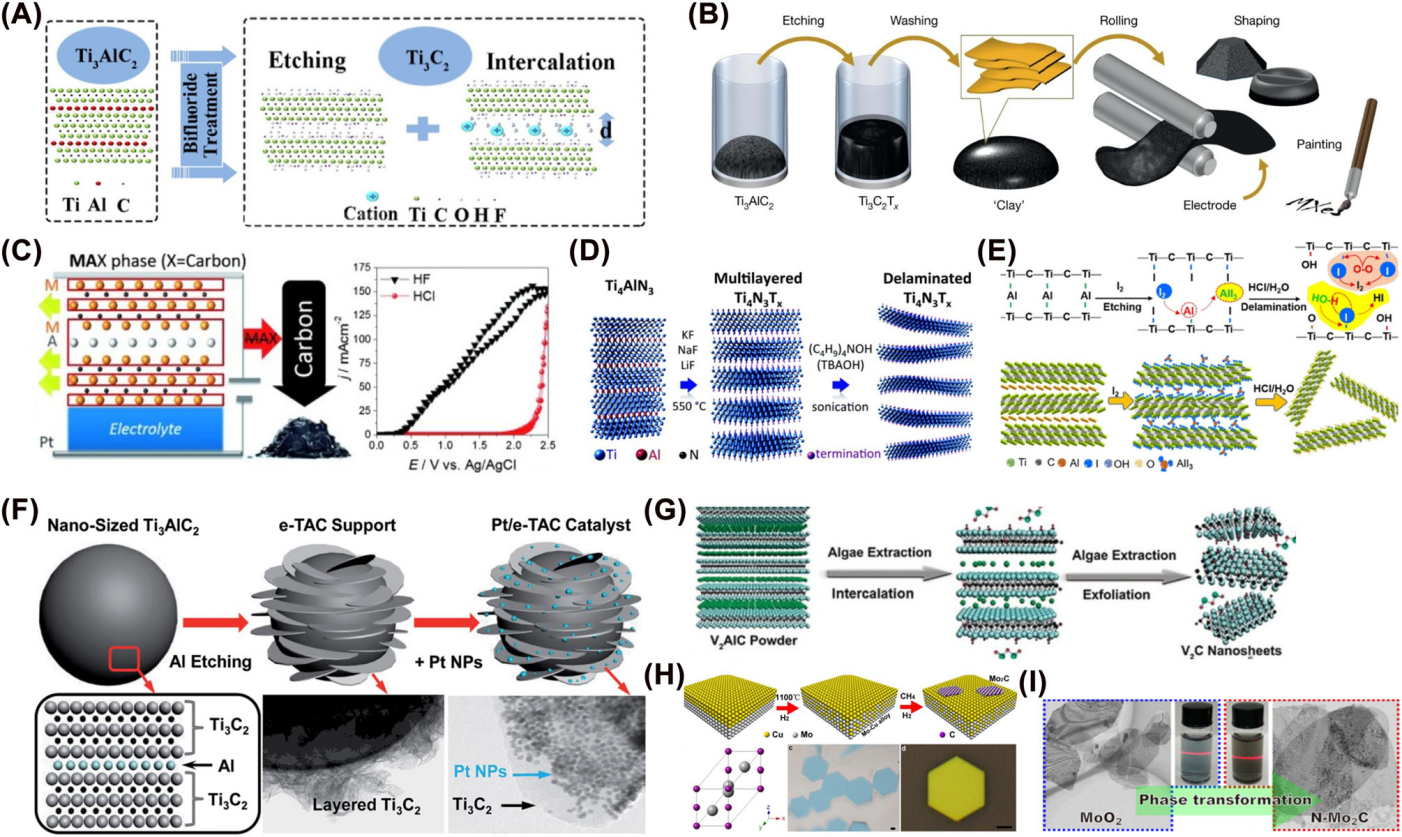
Preparing of MXenes. “Top–down” method: (A) HF selectively etches metal layers. (B) Acid/fluoride etching. (C) Carbide-derived carbon is formed by the electrochemical etching method. (D) Al atomic layer was etched by molten fluoride salt under argon at 550 °C. (E) Iodine-assisted strategy for etching Ti_3_AlC_2_ in anhydrous acetonitrile. (F) Ar/H_2_ thermal reduction strategy to produce TiC MXenes. (G) The algae extract to etch V_2_AlC crystals in an aqueous solution. “Bottom–up” method: (H) Mo_2_C crystals on the liquid copper surface by ambient pressure CVD. (I) synthesize N-doped Mo_2_C nanosheets based on the MoO_2_ template. Figures are adapted from references [6, 29–36].

HF selectively etches metal layers, mainly adopting the following equation of reaction to complete the preparation of MXenes (Figure 2A). The first non-MAX phase precursor synthesized Mo_2_CT_*x*_ was Mo_2_Ga_2_C by HF etching of Ga [28]. HF etching method has the advantages of simple operation and low reaction temperature and is suitable for etching the MAX phase containing Al and part of non-MAX phases. However, it suffers from the disadvantages of high corrosivity, toxicity, operational risk, and poor energy storage [25].

(1) $$M_{\text{n}+1}AlX_{\text{n}}+3HF\to M_{\text{n}+1}+AlF_{3}+1.5H_{2}$$

(2) $$M_{\text{n}+1}X_{\text{n}}+2H_{2}O\to M_{\text{n}+1}X_{\text{n}}{\left(OH\right)}_{2}+H_{2}$$

(3) $$M_{\text{n}+1}X_{\text{n}}+2HF\to M_{\text{n}+1}X_{\text{n}}F_{2}+1.5H_{2}$$

#### 3.1.3 Acid/fluoride etching

Halim et al. made the first hydrogen fluoride salt etching application using NH_4_HF_2_ sputter-deposited epitaxial Ti_3_AlC_2_ films [13]. Owing to the change of fluoride salt can adjust the interlayer spacing of MXenes, Ghidiu et al. conducted an etching process with HCl/LiF solution at 40 °C (Figure 2B) [29]. Additionally, this synthesis strategy can etch Ti_3_AlC_2_ without water and apply to water-sensitive MXenes materials. Difluoride salts are solid at room temperature and much safer than HF, and more attention should be paid for their application to exfoliate MAX phases. Multilayer Ti_3_C_2_T_*x*_ MXenes material reported by Wu et al. was etched by a hydrothermal process using oxalic acid and NH_4_F at different temperatures of 100–180 °C for 24 h (Figure 2C) [30]. The kinetic of this method depended on the acidity of the dissociated organic anion and its interaction with the dissociated F of the ionic liquid.

#### 3.1.4 Electrochemical etching method

The metal-carbon atoms are selectively extracted from the ternary layered carbides in an electrochemical manner. The carbide was added in a NaCl, HCl, or HF solution, and an anodic potential was applied so that carbide-derived carbon (CDC) is formed (Figure 2D), and a carbon film with a very narrow pore size distribution was formed [31]. The critical points of this method were the voltage, etching time, and electrolyte concentration. However, this method was unsuitable for large-scale preparation due to its low yield.

#### 3.1.5 Molten salt method and other methods

Carbide MXenes can be easily and successfully prepared by HF or fluoride etching, but nitride MXenes cannot be prepared. The Ti–Al bond in Ti_*n*+1_AlN_*n*_ is stronger than that in Ti_*n*+1_AlC_*n*_, so the preparation of Ti_*n*+1_AlN_*n*_ requires higher energy. Meanwhile, Ti_*n*+1_AlN_*n*_ is less stable and easy to degrade in HF. The molten salt method uses low melting point salt as flux with improved ion diffusion rate. Urbankowski et al. reported the first Ti_4_N_3_-based MXenes, using the molten fluoride salt for etching an Al atomic layer at 550 °C under argon conditions [37]. Recently, other new synthetic methods have also been explored. For example, halogen can also be used as an etchant to de-etch the MAX phase. Shi et al. designed an iodine-assisted way for etching Ti_3_AlC_2_ in anhydrous acetonitrile (Figure 2E) [32]. Mei et al. reported a new Ar/H_2_ thermal reduction strategy to produce TiC MXenes from the sulfur Ti_2_SC MAX phase (Figure 2F) [33]. Zada et al. used algae extract to etch bulk V_2_AlC crystals in an aqueous solution (Figure 2G) [34]. It has also been reported using UV-induced etching [38] and surface acoustic waves for ultrafast one-step synthesis of MXenes [39].

#### 3.1.6 Bottom-up

The bottom-up method for preparing MXenes is a chemical synthesis that includes CVD, atomic layer deposition (ALD), plasma-enhanced pulsed laser deposition (PEPLD), template methods, etc. Xu et al. produced ultrathin α-Mo_2_C crystals of several nanometers by using methane as a carbon source, copper foil on Mo foil as substrate, and a temperature higher than 1085 °C [40]. Geng et al. reported the growth of Mo_2_C crystals with controllable thickness and morphology on a liquid copper surface by ambient pressure CVD (Figure 2H) [35]. ALD, a variant of CVD, is a gas-phase method based on two successive self-limiting surface reactions. However, the rate of final products is usually low and requires special equipment. Zhang et al. demonstrated a type of Mo_2_C thin films with controlled crystal structure growth on sapphire substrates by PLD at a temperature of 700 °C [41]. Jia et al. used MoO_2_ as a template that was also a highly active electrocatalyst to synthesize N-doped Mo_2_C nanosheets (Figure 2I) [36]. Compared with the top–down method, the bottom-up approach saves raw materials and accurately controls the element composition, size, and surface groups. However, it is a challenge to prepare large-size MXenes. Most of the reported MXenes are prepared by the top-down method, with few reports on the bottom–up approach.

### 3.2 Surface modification

By altering the surface and interlayer spacing of MXenes, the proton transport efficiency will be enhanced, which will be beneficial to the electrochemical properties of MXenes. MXenes can be doped with various elements, nanoparticles, ligands, drugs, and other surface modifications to obtain desired characteristics. Doping with Fe^3+^, Co^2+^, Ni^2+^, Mn^2+^, and other metal ions is helpful to promote intrinsic performance. Zhang et al. replaced a Cu layer electrodeposited thereon by a Pt current using Pt-modified SnO_2_C (Pt/SnO_2_C) nanofibers. Thus, SnO_2_C nanofibers exhibited competitive oxygen reduction reaction catalytic activity, enhanced methanol tolerance, and superior durability [42]. Cao et al. utilized Au nanocrystals to selectively grow on the edges of TiO_2_ nanosheets with highly exposed (001) facets to fabricate Au-TiO_2_ NSs as an acoustic sensitizer [43]. Polymers such as dextran, cellulose, chitosan, polyethylene glycol (PEG) [44–46], polyethyleneimine (PEI) [47], polyvinylpyrrolidone (PVP) [48], polyacrylic acid, and polyvinyl alcohol [49], which are used to improve the stability, hydrophilicity, degradability, and biocompatibility of the MXenes. Pan et al. prepared Gd^3+^-doped MoSe_2_ nanosheets by a simple liquid phase method with a PEG modification on the surface for better PAI [50]. Cao et al. used vanadium carbide quantum dots (V_2_C QDs) with an engineered exosome (Ex) carrier to accomplish effective tumor therapy via bio-membrane modification [51]. MXenes decorated with metal nanoparticles can exhibit a strong plasmon-photothermal effect. By modifying with biomolecular such as folic acid [52], HA, arginyl glycyl aspartic acid, and chlorophyll [45], endowing MXene materials with excellent biocompatibility, strong physiological stability, and high clinical transformation potential. Radioisotope-based modified MXenes such as^131^I, and ^64^Cu can be adapted for imaging-guided cancer treatment [53, 54]. Overall, MXenes can be adjusted by different compositions, sizes, thicknesses, controlled surface functional groups, and surface terminals, confer different characteristics, and can be applied in biomedical fields [55].

## 4 Biosensing application

### 4.1 MicroRNA detection

As the Mxenes materials process unique metallic conductivity and hydrophilic properties which are fit for microRNA (miRNA) analysis, they are able to adsorb the single-stranded DNA (ssDNA) through weak Van Der Waals force. The biosensor based with fluorescent signals (FL), surface-enhanced Raman spectroscopy (SERS), photoelectrochemical (PEC) and electrochemical (EC) have been exploited [56] (Table 1).

**Table 1:** 2D MXenes applied for microRNA detection.

	MicroRNA	Samples	Detection method	Limit of detection	Reference
MXene/MoS_2_@AuNPs	miRNA-182	Human serum	SERS	6.61 am	[57]
Ti_3_AlC_2_-Au	miR-377	Human serum	EC	1.35 aM	[58]
Ti_3_C_2_T_*x*_ QDs/(001) TiO_2_/FTO	miR-155	/	PEC	25 fM	[59]
Ti_3_C_2_@ReS_2_	miRNA-141	Human serum	PEC	2.4 aM	[60]
FWNs	miR-21/miR-210	H1299	FL	0.75 nM	[61]
AuNP@MXene/Au	miR-141/miR-21	Human plasma	EC	204 aM/138 aM	[62]
Mo_2_C	miR-21	/	EC	0.34 fM	[63]
Mo_2_C QDs	miR-21	B16-F10/A549/MDA-MB-231	FL	/	[64]
Ti_3_C_2_	miR-141	/	EC	0.26 pM	[65]
Co-MOF-ABEI/Ti_3_C_2_T_*x*_	miR-21	/	EC	3.7 fM	[66]
Luminol-Au NPs-Ti_3_C_2_	miR-155	Human plasma	EC	0.15 fM	[67]
GSH-MQDs	miRNA-221	/	EC	10 fM	[68]

**Figure 3:**
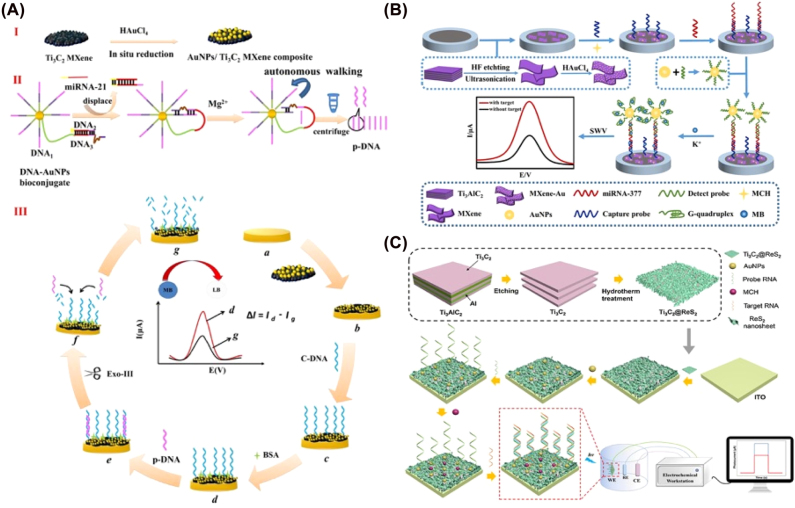
Application of MXenes in microRNA detection. (A) Gold nanoparticles (AuNPs)-decorated Ti_3_C_2_ MXenes served as an electrochemical biosensor. By combining with a cascaded signal amplification strategy, miRNA-21was sensitivity detected. (B) Ti_3_AlC_2_-Au nanocomposites and G-quadruplex nano-amplification based electrochemical biosensor. (C) Ti_3_C_2_@ReS_2_ based biosensor. Figures are adapted from references [58, 60, 70].

In the presence of miRNA, the duplex DNA will be formed and dissociated from the surface of the Mxene materials. The fluorescence will be recovered that realized rapid, simple, and selective recognition [69]. In Wang’s work, a synergistic calibrated SERS strategy based on MXene/MoS_2_@AuNPs with controllable morphology has been presented for detecting miRNA-182. The system has three characteristic Raman peaks (at 382 cm^−1^ and 402 cm^−1^ corresponding to MoS_2_ and at 611 cm^−1^ corresponding to MXene) as a benchmark instead of additional beacon molecules. Specifically, the LOD was 6.61 am for miRNA-182 in human serum samples [57]. Various studies focused on signal amplification methods to enlarge the detection sensitivity and accuracy. For example, Wang et al. fabricated novel *in situ* reductions of gold nanoparticles (AuNPs)-decorated Ti_3_C_2_ MXene electrochemical biosensor combined with a cascaded signal amplification strategy for the detection of miRNA-21, MXene served as both the reductant and stabilizer. By cascaded signal amplification, the assessment indicated that this electrochemical biosensor has a detection limit of 50 aM (*S*/*N* = 3) (Figure 3A) [70]. Li’s group also constructed a Ti_3_AlC_2_-Au nanocomposite and G-quadruplex nano-amplification-based electrochemical biosensor for miRNA-377 measurement in human serum samples. Specifically, the designed biosensor displayed excellent sensing performance with a limitation of detection (LOD) as low as 1.35 aM (Figure 3B) [58]. For microRNA-155 detection, a Ti_3_C_2_T_*x*_ QDs/(001) TiO_2_/FTO platform was constructed. Detailed, the structure was composed of TiO_2_ and Ti_3_C_2_T_*x*_ QDs by a type II heterojunction and the LOD was 25 fM [59]. Xu’s group presented a Ti_3_C_2_@ReS_2_ via the vertical anchoring flaky ReS_2_ on the Ti_3_C_2_ backbone for miRNA-141 detection. According to the assessment, the Ti_3_C_2_@ReS_2_ sample containing 45 wt% of ReS_2_ showed a 2.48-time promotion in the photocurrent compared to ReS_2_ owing to the synergistic effects of its photoactive and conductive counterparts. In essence, the estimated LOD was 2.4 aM (Figure 3C) [60].

**Figure 4:**
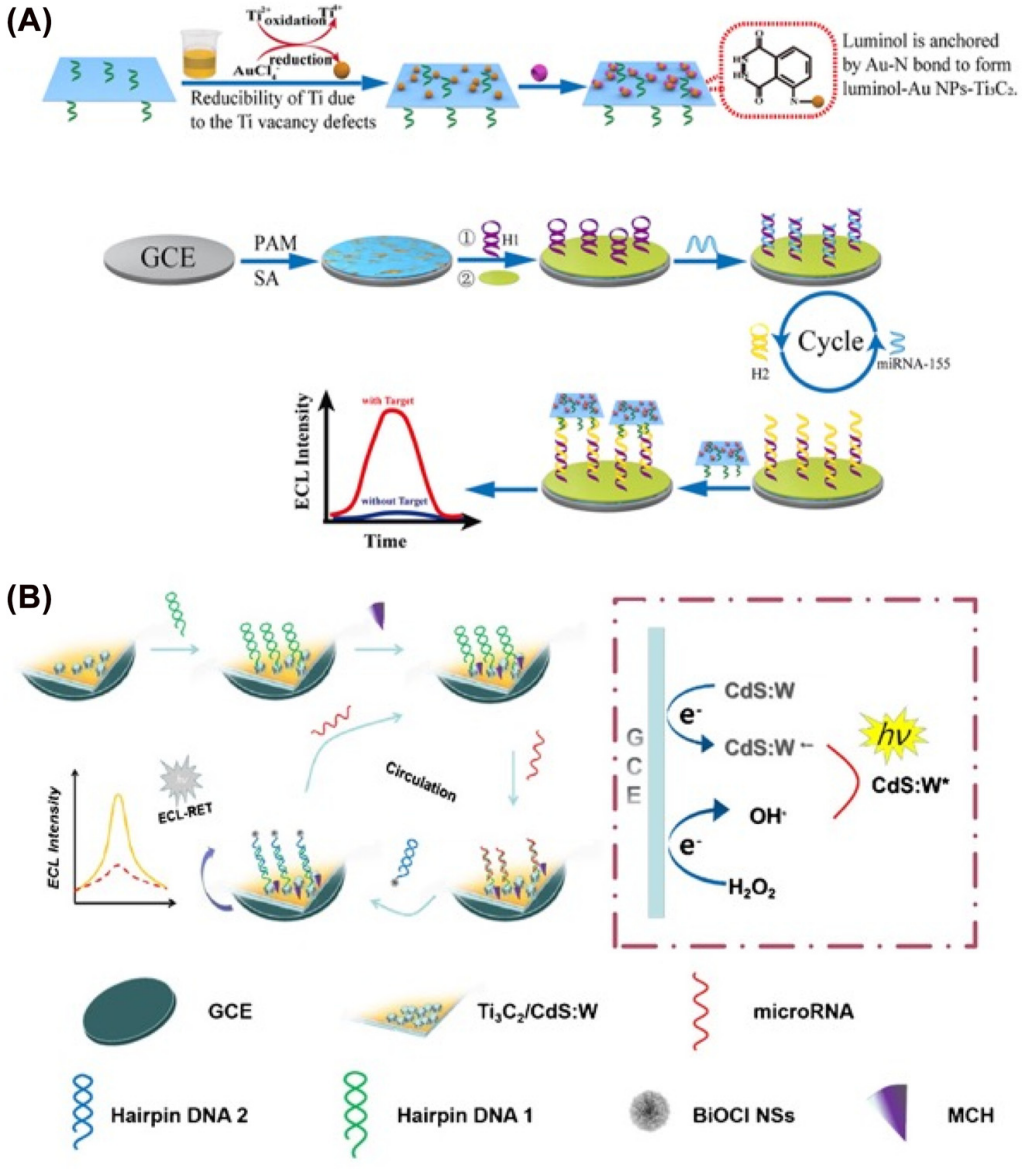
Mxenes material-based ECL biosensor. (A) Luminol-Au NPs-Ti_3_C_2_ as an ECL biosensor for miRNA-155 detection (B) GSH-MQDs biosensor for the detection of miRNA-221 and magnetic biomimic vesicles. Figures are adapted from references [67, 68].

There are also numerous examples of the detection of multiple intracellular miRNAs. Liao et al. used folate-adsorbed carbon nitride to create a multipurpose probe for *in-situ* monitoring of various miRNAs [71]. Lee et al. synthesized AuNP@MXene/Au to modify with vast numbers of DNA probes for miRNA-21 and miRNA-141 detection, and LOD was determined as 204 aM and 138 aM, respectively. Moreover, this device successfully indicated three cancer plasma samples [62]. Tian et al. reported a simple amplification strategy of enzyme-free miRNA target-triggered strand displacement reaction to fabricate a molybdenum carbide (Mo_2_C) biosensor with ferrocene to detect miR-21 [63]. Additionally, Dai et al. also synthesized a class of monolayer Mo_2_C QDs with biocompatibility and water solubility by liquid exfoliation method to deliver the optimized molecular beacons MB into cells for accurate quantitative detection of mature miRNAs [64]. Wang et al. reported a stable luminol-Au NPs-Ti_3_C_2_ as an ECL biosensor for miRNA-155 detection. The immobilization of ECL emitters is a versatile strategy that not only decreases the electron transmission distance, but significantly improves the ECL signal of luminol. The LOD was 0.15 fM in human serum samples (Figure 4A). Ma et al. constructed a GSH-MQDs biosensor for the detection of miRNA-221 and magnetic biomimic vesicles. Glutathione is used as a precursor to improving the oxidation resistance of MXene effectively. Both the metal atoms of the MXene and the sulfhydryl group of GSH could reduce the defects in the MXene-derived QDs. Furthermore, on the cyclic amplification with a T7 exonuclease, the biosensor can detect miRNA-221 in the triple-negative breast tumor tissues (Figure 4B). Du et al. used CdS: W nanocrystals modified Ti_3_C_2_ MXenes as an ECL signal emitter to detect miRNA-141 [65]. Jiang et al. reported a hybrid luminescent Co-MOF-ABEI/Ti_3_C_2_T_*x*_ composite used to estimate miRNA-21 with a detection limit of 3.7 fM [66]. Overall, compared with FL output signals, the biosensor with EC and PEC exhibited much higher sensitive performance.

### 4.2 Gas detection

**Figure 5:**
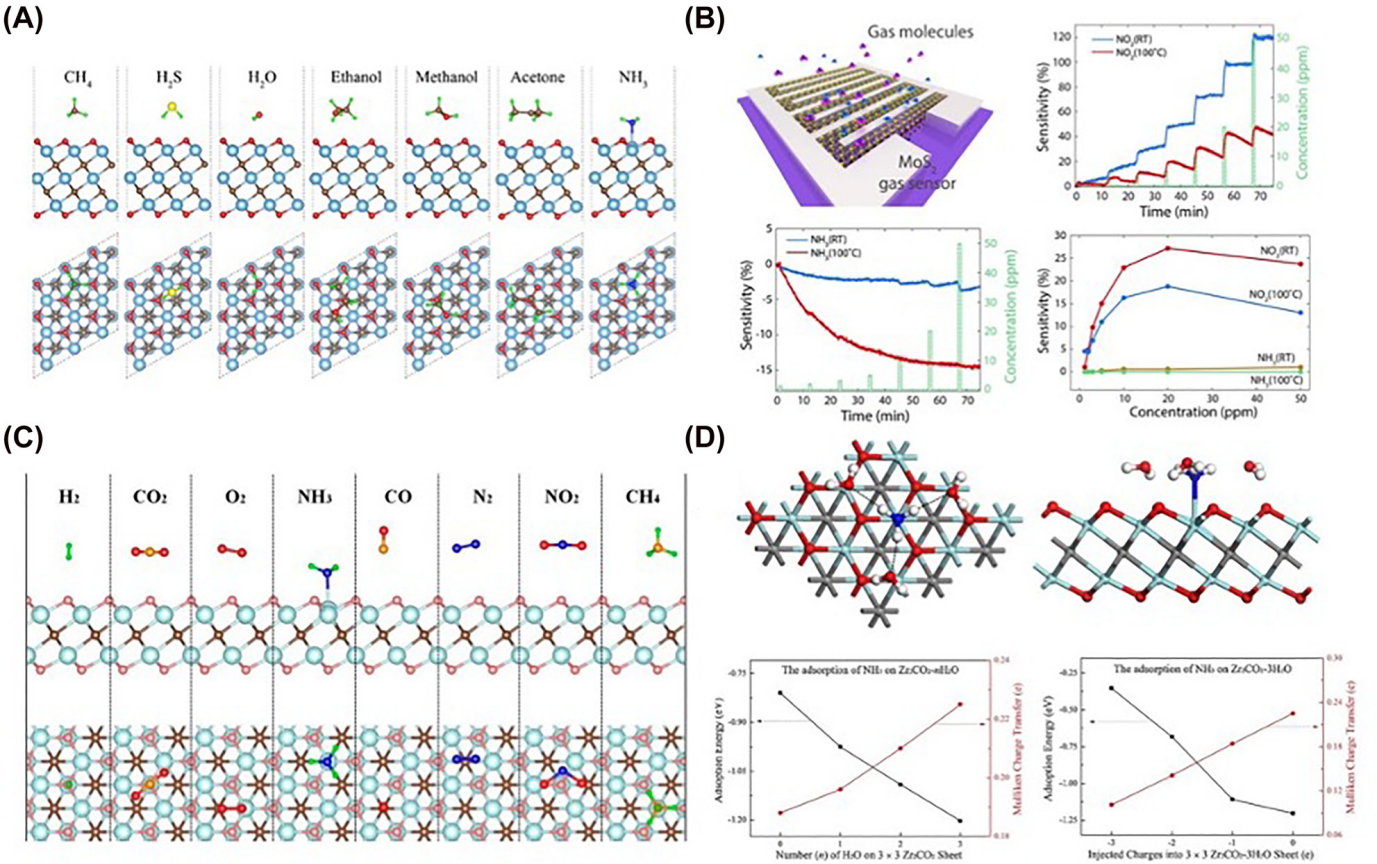
Application of MXenes in gas small molecule detection. (A) Ti_3_C_2_ MXene based gas sensor for NH_3_ detection. (B) Gas sensor based on MoS_2_ synthesized by CVD for detecting NO_2_ and NH_3_. (C) Porous DPSNs@X% TiO_2_-X composite photocatalyst was used for the detection of NH_3._ (D) O-terminated semiconducting MXenes are excellent materials for NH_3_ sensors or capture. Figures are adapted from references [73, 80, 84, 85].

MXenes have good sensitivity to many small molecules and can be used for gas sensing [72]. Wu et al. reported a Ti_3_C_2_ MXene based gas sensor for NH_3_ detection with high selectivity (Figure 5A) [73]. Many other MXenes such as SnO [74], CuO [75], NiO [76], In_2_O_3_ [77], WO_3_ [78], and Co_3_O_4_ [79] exhibit good characteristics for gas analysis. Cho et al. designed a MoS_2_ based on CVD for detecting NO_2_ and NH_3_ (Figure 5B) [80]. In addition to MoS_2_, other metal disulfides such as MoSe_2_, SnS_2_, and WS_2_ also have applications in gas detection [81–83]. Yu et al. studied the adsorption of NH_3_, H_2_, CH_4_, CO, CO_2_, N_2_, NO_2_, and O_2_ by monolayer Ti_2_CO_2_; only NH_3_ could be chemisorbed on Ti_2_CO_2_ (Figure 5C) [84]. Xiao et al. considered the interaction between NH_3_ and O-terminated semiconductor MXenes (M_2_CO_2_, M = Sc, Ti, Zr, Hf) with different charge states by using first-principles simulations. NH_3_ can strongly adsorb on M_2_CO_2_ with obvious charge transfer. O-terminated semiconductor MXenes are excellent materials for NH_3_ sensors with the advantage of highly reversible release and capture (Figure 5D) [85].

### 4.3 Detection of other small molecules

**Figure 6:**
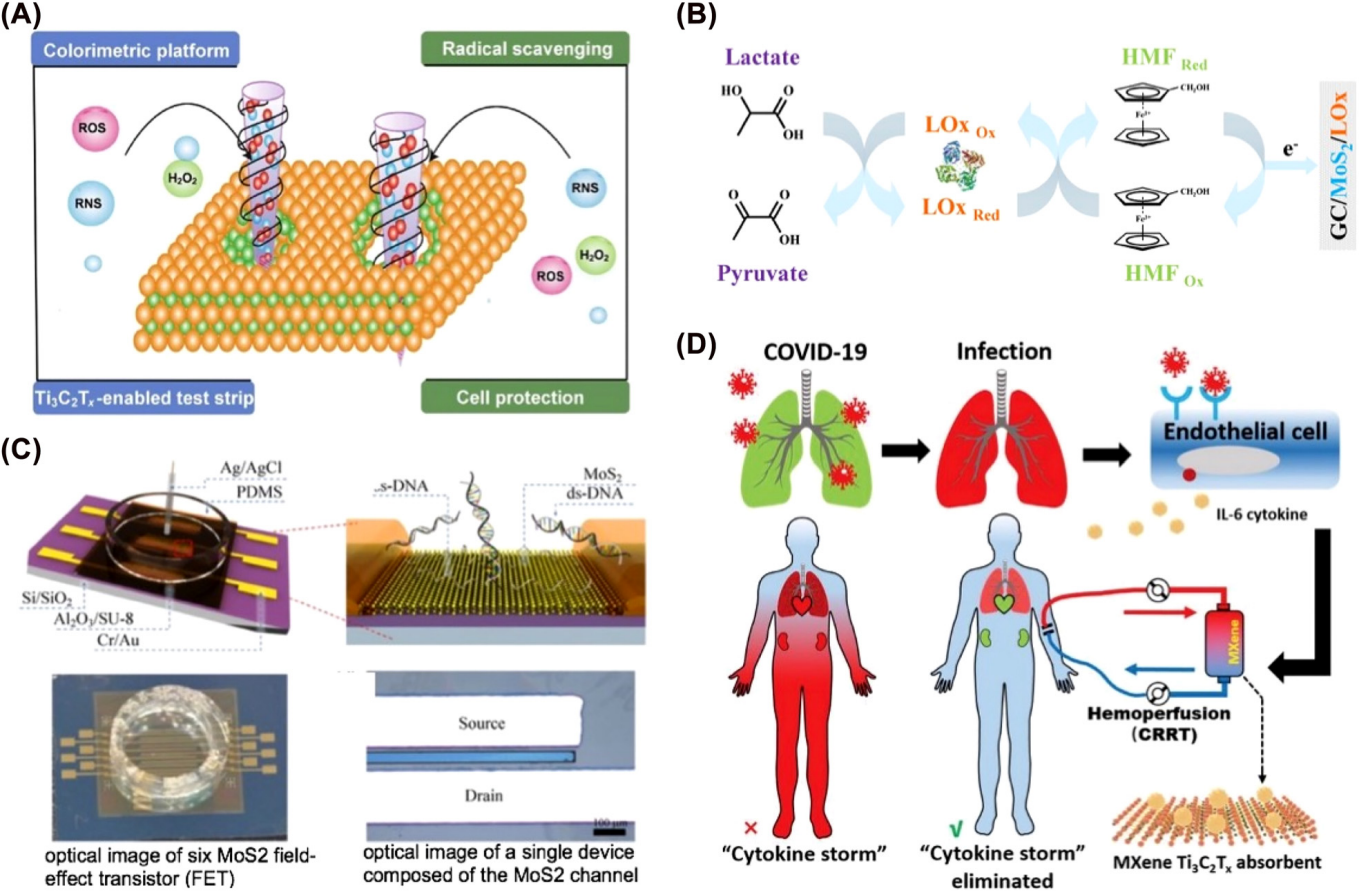
Application of MXenes in the detection of small molecules. (A) MXene-Ti_3_C_2_ nanosheet as a colorimetric strip for reactive oxygen species (ROS) and reactive nitrogen species (RNS) detection and scavenging. (B) Lactate detection sensor. (C) MXenes sensor detects specific hybridization with probe DNA molecules based on molybdenum disulfide nanosheets and lactate oxidase. (D) Ti_3_C_2_T_*x*_ is an ultra-efficient hemoperfusion absorber for eliminating cytokines. Figures are adapted from references [86, 88, 96, 97].

The qualitative and quantitative analysis of metabolites is a crucial indicator of physiological and pathological alterations. H_2_O_2_ is an essential molecule in many signal transduction processes *in vivo* and is involved in cellular metabolism. MXene-Ti_3_C_2_ nanosheet was designed as a colorimetric strip that reacted with H_2_O_2_ and has good performance for free radical scavenging (Figure 6A) [86]. Ti_3_C_2_T_*x*_/PtNP sensor can detect small redox molecules such as ascorbic acid, dopamine, uric acid, and acetaminophen [87]. MoS_2_ nanosheets and lactate oxidase biosensors can selectively detect lactate (Figure 6B) [88]. Moreover, MXenes materials can detect glucose, GSH, proteins, dengue DNA and RNA, circulating tumor DNA, and T4 polynucleotide kinase (T4 PNK) [89–95]. For example, MoS_2_ could specifically detect a minimal amount of target DNA molecules (Figure 6C) [96]. Very recently, Wang et al. reported Ti_3_C_2_T_*x*_ as an ultra-efficient hemoperfusion absorber for eliminating the cytokine storm syndrome which induced by COVID-19. The molecular-level investigations demonstrated that Mxenes has strong chemisorption for immobilizing cytokine interleukin-6 and good blood compatibility (Figure 6D) [97].

## 5 Biological imaging and disease diagnosis

MXenes have great potential for the non-invasive imaging, including MRI, positron emission tomography (PET), X-ray computed tomography, FL, PAI, etc. [98–105]. Nanoscale MXenes are also suitable for bioimaging and disease diagnosis [106, 107]. First, the nanoscale size enables them to exist in the organism for a longer time, effectively avoiding the self-clearing function of the blood circulation, enabling intermolecular interactions, and inducing luminescence. Secondly, certain hydrophilic functional groups on the surface of MXenes could improve water solubility and have promising biological. Especially, recent studies have shown that MXenes can be degraded and eliminated in organisms. In addition, the near-infrared absorption of MXenes makes them a suitable contrast agent for PAI.

### 5.1 Photoacoustic imaging

As a new diagnostic imaging technique, PAI induces optical imaging by irradiating the tissue with excitation light. Because of its low tissue attenuation coefficient, PAI can achieve the purpose of real-time detection of biological lesions. The spectrum of MXenes extends from ultraviolet-visible to NIR, and MXenes have good photothermal conversion capability, which enables them to be effective PAI contrast agents. The photothermal effect has been demonstrated in various MXene compositions such as Ti_3_C_2_, Nb_2_C, and Ta_4_C_3_ [36, 108, 109]. The strong absorption spectrum is the NIR region, facilitating for deep tissue PAI. For example, Chen et al. fabricated niobium carbide (Nb_2_C) MXene via a simple and scalable two-step liquid exfoliation method for efficient *in vivo* photothermal ablation of NIR-II windows in mouse tumor xenografts with good PAI ability [36]. Dai et al. developed Mo_2_C QDs by a simple ultrasound-assisted liquid phase exfoliation method showing excellent performance in PAI [110]. The Mo_2_C QDs have high stability, biocompatibility and low cytotoxicity.

### 5.2 SERS and fluorescence imaging

**Figure 7:**
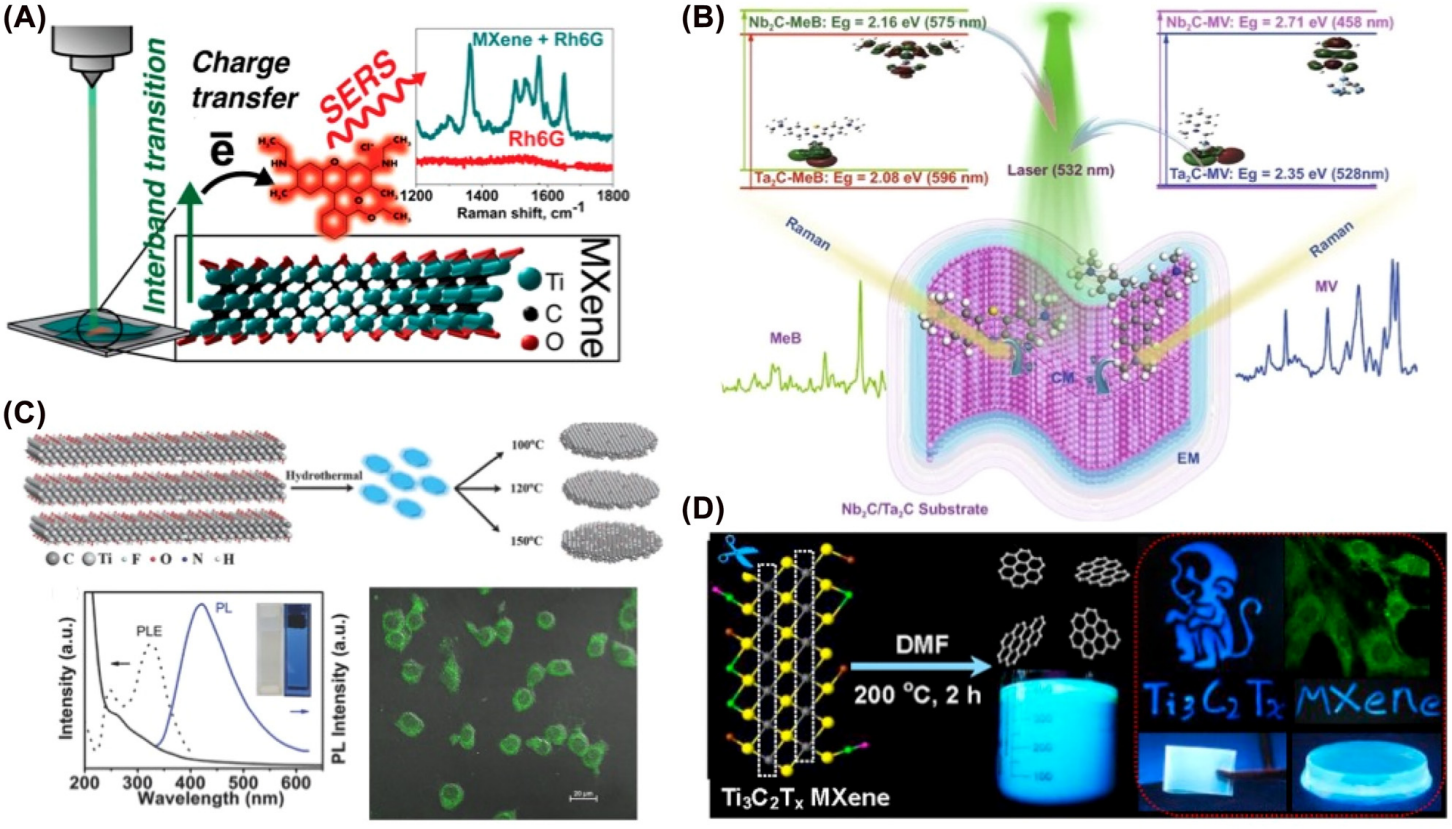
Application of MXenes in bioimaging. (A) Ti_3_C_2_T_*x*_ has enhanced the Raman signal. (B) Nb_2_C and Ta_2_C MXenes are remarkable SERS enhancement probes. (C) MQD showed excitation-dependent photoluminescence spectra with high quantum yields. (D) Photoluminescence Ti_3_C_2_ MXene quantum dots for multicolor cell imaging. Figures are adapted from references [113–116].

The LSPR effect of MXene nanosheets with semi-metallic properties can enhance the Raman scattering signal and serve as a good building block for SERS. Emitting MXene QDs are reported by fabricating small-size dot phase MXenes. Strong emissions were realized under specific wavelength excitation, which resulted from the quantum confinement caused by size effect and luminescence resulting in induced defects [111, 112]. Based on this, many researchers used MXene SERS imaging for sensitive detection applications. For instant, the Yury Gogotsi group reported titanium carbide MXene Ti_3_C_2_T_*x*_ that can enhance Raman signal from organic dyes on a substrate and in solution (Figure 7A) [113]. Due to the synergistic effect of the charge transfer resonance and electromagnetic enhancement, Nb_2_C and Ta_2_C MXenes were shown as remarkable SERS enhancement probes for sensitively detecting the SARS-CoV-2 spike protein (Figure 7B). The results indicated that the detection limit is as low as 5 × 10^−9^ M, which is suitable for achieving real-time monitoring and early warning of novel coronavirus [114].

With the recently developed luminescent MXene QDs, MXenes have been applied to luminescent cell imaging. Similar to graphene and carbon dots, MXene QDs exhibited luminescence properties, and their emission is related to excitation. For example, Zhi et al. developed photoluminescent Ti_3_C_2_ MXene QDs (MQD) for multicolor cell imaging. Due to the strong quantum confinement, the as-prepared MQD showed excitation-dependent photoluminescence spectra with high quantum yields. The application of MQD as a biocompatible multicolor cell imaging probe and zinc ion sensor was demonstrated in (Figure 7C) [115]. Wang et al. synthesized amphiphilic carbide-derived GQDs combined with layered Ti_3_C_2_X MXene to apply in cellular imaging due to their excellent properties, such as bright and tunable photoluminescence, low cytotoxicity, good photostability, and chemical inertness (Figure 7D) [116].

### 5.3 Multifunctional MXenes theranositic platform

**Figure 8:**
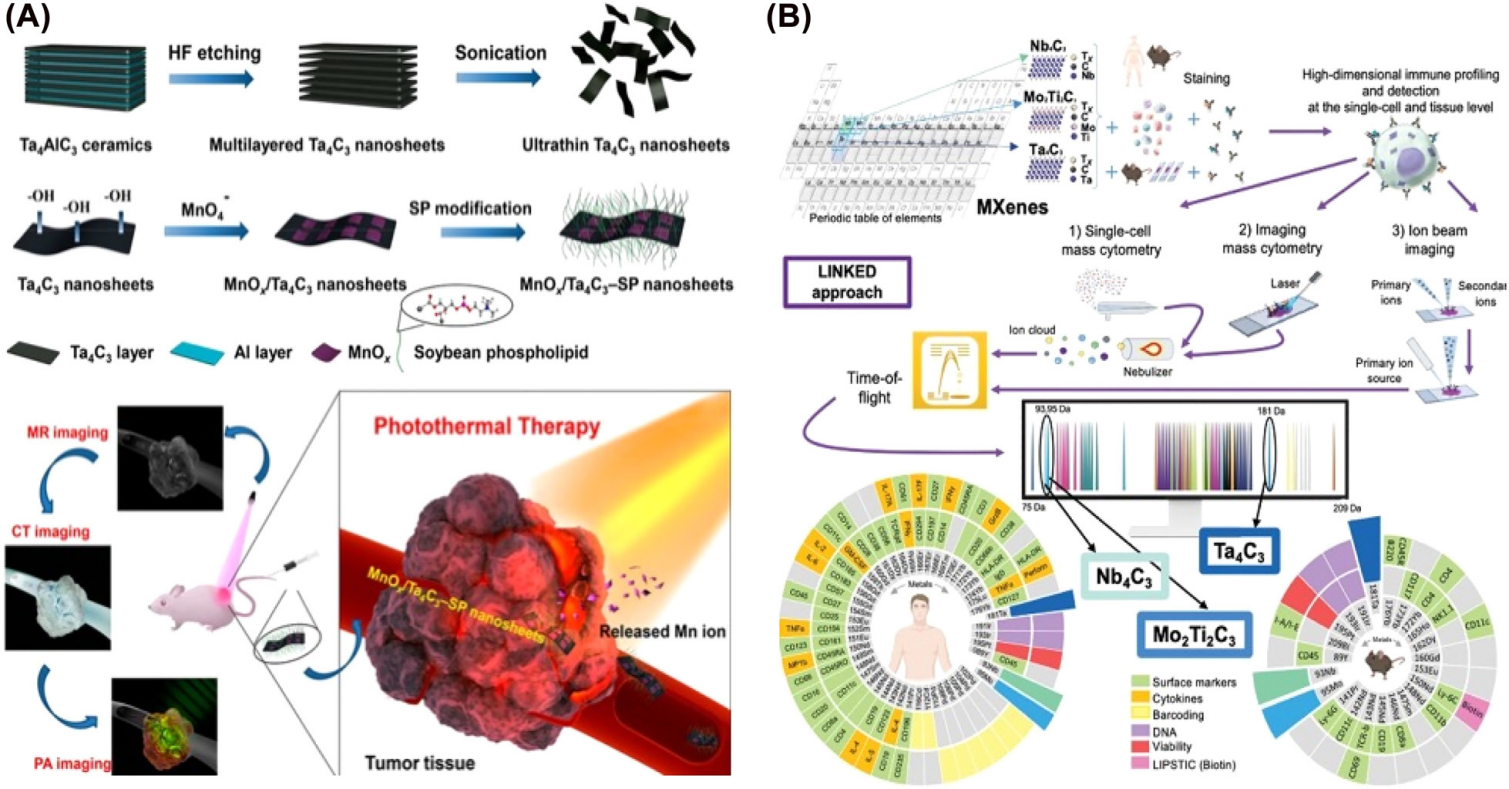
MXenes for therapeutic and diagnostic multimodal imaging applications. (A) MnO*x*/Ta_4_C_3_ were the high-performance contrast agents for CT, T1-weighted MRI, and PAI. (B) New biomedical application based on Nb_4_C_3,_ Mo_2_Ti_2_C_3,_ and Ta_4_C_3_ for detecting cells and tissues using three mass-cytometry-based methods (single-cell mass cytometry, imaging mass cytometry, and ion-beam imaging). Figures are adapted from references [121, 125].

MXenes-based MXenes theranositic platform could be used for imaging localized tumors, tracking drug delivery, and monitoring cancer treatment. Owing to their large surface area, MXenes can adsorb different imaging molecules and nanoparticles, such as fluorophores, gadolinium, radioactive elements, IONP, and other NPs [19, 109, 117], [118], [119]. For example, Ti_3_C_2_T flakes were covalent functionalized with a chelating agent diethylenetriaminepentaacetic acid (DTPA), and then complexed with Gd^3+^ ions for *T*_1_ MR imaging [120]. Wu’s group constructed the tantalum carbide (Ta_4_C_3_) Mxene functionalized with manganese oxide nanoparticles (MnO_*x*_) component for multiple imaging-guided photothermal tumor ablations. The MnO_*x*_/Ta_4_C_3_ has achieved high-performance contrast agents for contrast-enhanced computed tomography, *T*_1_-weighted MRI, and contrast-enhanced PAI (Figure 8A) [121]. Various biocompatible nanoplatforms formed from Ti_3_C_2_, Ta_4_C_3_, and Nb_2_C nanosheets are suitable for diagnosis/imaging. However, compared to the carbide- and carbonitride-based MXenes, nitride-based MXenes have been rarely explored, especially for biological and biomedical appliances [122, 123]. Actually, these nitride-based MXenes exhibited better biodegradability in the physiological environment. According to a facile top-down method, Prof. Huang’s group synthesized titanium nitride quantum dots (Ti_2_N QDs) in solution. The Ti_2_N QDs exhibited good performance on PAI-guided PTT in both NIR-I/II biowindows for precision cancer treatment [124]. Interestingly, MXenes with enzymatic labels were used to fabricate a versatile multiplexed label-free single-cell detection strategy with high-dimensional imaging. Generally, a set of MXenes is selected to ensure mass detection within the cytometry range while avoiding overlap with more than 70 currently available tags and able to survey multiple biological parameters at the single-cell level or in different organs (Figure 8B) [125].

## 6 Conclusion and outlook

MXenes offer unique properties and enormous potential in biological applications. However, the clinical translation of these compounds still confronts several obstacles. Most of MXenes are prepared by the top–down method, lacking an approach to precisely control the size, layer number distribution, and surface groups of the final products. Large-scale preparation is essential for further commercial applications. However, the current synthesis of MXenes is only in the laboratory stage. There are few bottom–up approaches, developing an effective bottom–up method for MXene synthesis is extremely desirable. In addition to nanosheets, it’s also important to construct MXenes with various morphologies such as nanotubes and nanocages. Furthermore, the combination of MXenes with other functional materials to form hybrid materials with attractive properties is also highly needed. Finally, the biosafety remains a key challenging issue. Scientists have conducted short-term toxicity and organ residue distribution studies to confirm that MXenes have excellent ideal short-term biosafety. However, long-term biosafety including genotoxicity, immunotoxicity, and reproductive toxicity are required. We believed that the development of chemical materials science, biology, and medicine and the collaboration among various disciplines would accelerate the bioanalytical and imaging applications of MXenes in the future.
